# Oral sex practices among men who have sex with men and transgender women at risk for and living with HIV in Nigeria

**DOI:** 10.1371/journal.pone.0238745

**Published:** 2020-09-04

**Authors:** Sarah J. Robbins, Wuese Dauda, Afoke Kokogho, Nicaise Ndembi, Andrew Mitchell, Sylvia Adebajo, Charlotte A. Gaydos, Sheila Peel, Habib O. Ramadhani, Merlin L. Robb, Stefan D. Baral, Julie A. Ake, Man E. Charurat, Trevor A. Crowell, Rebecca G. Nowak, on behalf of the TRUST/RV368 Study Group

**Affiliations:** 1 Institute of Human Virology, University of Maryland School of Medicine, Baltimore, MD, United States of America; 2 Institute of Human Virology Nigeria, Abuja, Nigeria; 3 HJF Medical Research International, Abuja, Nigeria; 4 U.S. Military HIV Research Program, Walter Reed Army Institute of Research, Silver Spring, MD, United States of America; 5 Maryland Global Initiatives Corporation- A University of Maryland Baltimore Affiliate, Abuja, Nigeria; 6 Johns Hopkins University School of Medicine, Baltimore, MD, United States of America; 7 Henry M. Jackson Foundation for the Advancement of Military Medicine, Bethesda, MD, United States of America; 8 Johns Hopkins School of Public Health, Baltimore, MD, United States of America; University of Westminster, UNITED KINGDOM

## Abstract

**Background:**

Men who have sex with men (MSM) and transgender women (TGW) are at risk for sexually transmitted infections (STIs), including those of the oropharynx. We estimated the prevalence and factors associated with oral sex practices and characterized oropharyngeal STIs among a cohort of MSM and TGW in Nigeria.

**Methods:**

From 2013 to 2018, TRUST/RV368 recruited MSM and TGW into HIV/STI diagnosis and treatment at community-based clinics in Nigeria. Participants who completed HIV testing and oral sex questions at enrollment were selected. Cross-sectional analyses with bivariate and multivariable logistic regression models estimated odds ratios (ORs) and 95% confidence intervals (CIs). Oropharyngeal swab testing for *Neisseria gonorrhoeae* (NG) and *Chlamydia trachomatis* (CT) began in 2014 and for those with diagnostic results at enrollment, the unadjusted association of oral sex practices with oropharyngeal STIs was conducted.

**Results:**

A total of 1342 participants had a median age of 25 years (interquartile range: 22–29), 58% were living with HIV, and 69% reported oral sex practices. Factors associated with increased odds of engaging in oral sex included living with HIV (adjusted [a]OR: 1.4, 95% CI: 1.1–1.8), self-identifying as a woman (aOR:1.8, 95% CI: 1.1–2.8), mobile phone ownership (aOR:2.3, 95% CI: 1.3–3.9), receptive anal sex (aOR:1.7, 95% CI:1.3–2.3) and multiple male sexual partners (2 to 4 vs. ≤1, aOR:1.5, 95% CI: 1.0–2.2; 5+ vs ≤1, aOR:2.9, 95% CI:1.9–4.3). Oropharyngeal STI prevalence was 7% (52/752) and higher among those who engaged in oral sex compared to those who did not (unadjusted OR: 2.5, 95% CI:1.2–5.3).

**Conclusions:**

Oral sex was common and associated with an increased odds of oropharyngeal STIs among MSM and TGW from Nigeria. In the absence of screening and treatment guidelines, condoms continue to be the mainstay for oral STI prevention. A pre-exposure prophylaxis for bacterial STIs would complement current prevention strategies to curb transmission.

## Introduction

Most prevention efforts for men who have sex with men (MSM) and transgender women (TGW) have focused on curtailing HIV with less emphasis on preventing transmission of other sexually transmitted infections (STIs) [1–3]. Previous studies, limited primarily to the United States, have suggested that condomless oral sex practices are common among MSM with less than 30% using condoms during oral sex [4–9]. In a prior study where participants reported 100% condom use during anal sex, 18% engaged in condomless oral sex [8]. Condomless oral sex allows transmission of STIs, such as gonorrhea, chlamydia, and human papillomavirus, through the oral mucosa. Oropharyngeal STIs among MSM range in prevalence (5–39%) depending on the infection, sample population and co-infection with HIV [10–13]. Little is known regarding the prevalence of oral sex practices and oropharyngeal STIs in TGW [13–15].

Across sub-Saharan Africa, oral sex practices broadly range from 8–84%, with nearly all studies focusing on heterosexual partnerships [4,16–18]. A small study among 42 MSM from Central African Republic found an overall prevalence of 67% and oral sex was more common among MSM-only as compared to men who have sex with men and women (MSMW) (100% vs. 59%, respectively) [4]. Furthermore, those living with HIV were significantly more likely to have regular receptive oral sex than those who were not living with HIV (86% and 23%, respectively) [4]. Other factors associated with oral sex practices globally included using a phone every day, internet use, low-risk perception of HIV, and recently being pregnant [17–21]. Additionally, having multiple sexual partners, anal sex, forced sex, and paid sex were also indicators of oral sex practices [17, 20]. However, many of these risk factors were derived primarily from large heterosexual studies [17, 18, 21] with only a couple from MSM [19, 20]. Whether these factors are consistent among MSM and TGW as compared to reproductive-aged adults is yet to be determined.

The TRUST/RV368 study is one of the largest cohorts of MSM and TGW in sub-Saharan Africa providing estimates of the overall burden of HIV and STIs in Nigeria. HIV prevalence ranged from 44–66% and incidence was 15 per 100 person-years [22, 23]. Prevalence estimates of *Neisseria gonorrhoeae* (NG) and *Chlamydia trachomatis* (CT) were high (26% and 14%, respectively) along with other STIs [24–26]. Moreover, participants of TRUST/RV368 reported incomplete condom uptake for HIV and STI prevention [27]. Therefore, TRUST/RV368 is well positioned to provide insights on oral sex practices and oropharyngeal STIs of MSM and TGW in Nigeria.

We hypothesized that participants living with HIV had a higher prevalence of oral sex than those who were not and this association would be modified by sexual practices with women. In addition, we evaluated whether oral sex practices were associated with oropharyngeal STIs among those with complete oropharyngeal laboratory testing results for NG and CT.

## Methods

### Study population and design

TRUST/RV368 recruited MSM and TGW between March 2013 and August 2018 through respondent driven sampling (RDS) to provide HIV/STI diagnosis and treatment at community-based clinics in Abuja and Lagos, Nigeria [28, 29]. In brief, initial “seed” participants were given 3 coupons to recruit 3 peers to begin a chain of recruitment. Eligibility criteria included age ≥16 years in Abuja and ≥18 years in Lagos, assigned male sex at birth, anal sex with a man in the past year, informed consent in English or Hausa, and a valid recruitment coupon. Age inclusion criteria differed between sites because of differences in institutional review board (IRB) recommendations. In Abuja, those aged 16–17 years were considered emancipated minors and exempt from parental consent. At enrollment, participants completed structured survey instruments collecting information on demographics and sexual practices, underwent physical examinations, and provided biological specimens for laboratory diagnosis of HIV and anogenital STIs. Oropharyngeal testing for STIs was initiated in October 2014 in Lagos and February 2015 in Abuja, about 1.5 to 2 years after the start of enrollment.

TRUST/RV368 was approved by the Nigerian Federal Capital Territory Health Research Ethics Committee, the Nigerian Ministry of Defense in Nigeria, the University of Maryland Baltimore, and the Walter Reed Army Institute of Research IRBs.

HIV status was assessed by a parallel testing algorithm, which included two rapid tests [Alere Determine (Waltham MA) and Trinity biotech Uni-Gold HIV (Wicklow, Ireland)] with discordant results undergoing a third rapid test, Chembio Diagnostics HIV-1/2 Stat Pak test (Medford NY). Voided urine and anal swabs were collected at each visit for diagnosis of urethral and rectal *Neisseria gonorrhoeae* (NG) and *Chlamydia trachomatis* (CT) using Aptima Combo 2 CT/NG Assay (Hologic, San Diego, CA) [28]. Oropharyngeal swabs collected after October 2014 were tested for pharyngeal NG and CT using the same diagnostic assays. All participants diagnosed with HIV or bacterial STIs were offered appropriate treatment according to the national guidelines for HIV and STDs [30].

### Exposure, outcome and covariates

The primary exposure variable was binary categorization of those living with or without HIV. The primary outcome was binary categorization of engaging in any oral sex practices. If a participant answered one or more men to the following question, “*In the last 12 months*, *how many men did you have oral sex with*?” or indicated oral sex based on frequency of condom use in the following questions, “*In the last 12 months*, *of the times you had oral sex with another man*, *how often was a condom used*?” then the participant was categorized as having any oral sex.

The primary covariates were derived from the literature or identified through directed acyclic graphs. These included mobile phone ownership, alcohol use, condom use during most recent anal sex act with a male partner, prior HIV testing, receptive and insertive anal sex, number of male anal sexual partners in the past 12 months, number of female vaginal sex partners in the past 12 months, and any transactional sex in the past 12 months where participants received money, drugs, food, shelter or transportation in exchange for anal or oral sex. Self-identified gender was categorized as man, woman, or non-binary. Non-binary included those who identified as “other” or “both man and woman”. Alcohol use was binary categorized no or yes if participants reported at least one drink in the last 30 days. The number of male anal sexual partners was categorized as 0–1, 2–4 and greater than 5 and number of female vaginal sexual partners was binary categorized as 0–1 or ≥2.

### Statistical analysis

Proportional distributions of demographic and behavioral characteristics by HIV and oral sex practices were evaluated using Pearson’s Chi-square test. The prevalence of oral sex and 95% confidence intervals (CIs) were estimated using a binomial distribution. Bivariate and multivariable logistic regression estimated odds ratios (ORs) with 95% confidence intervals (CIs). We evaluated female partners as an effect modifier (0 to 1 or ≥2) with bivariate logistic regression and Chi-square interaction p-value. To assess confounding, crude associations of the covariates with the exposure and outcome were conducted separately. Any covariates independently associated with the exposure and outcome (p<0.05) and altered the Beta coefficient more than 10%, literature-based *a priori* covariates, and covariates identified through directed acyclic graphs analysis were tested as potential confounders. To obtain the final multivariable model we used a backward stepwise approach to eliminate insignificant covariates (p<0.05) except for literature-based *a priori* covariates. Our final model assessed the odds of having oral sex among those living with HIV as compared to those who were at risk of HIV independent of other demographic and behavioral characteristics. Bivariate logistic regression was used to assess the crude association of oral sex and oropharyngeal STIs. In all analyses, any p-value <0.05 was considered significant. Statistical Analysis Software (SAS) version 9.4 (SAS Institute, Cary, NC) was used for cross-sectional data analysis.

## Results

Of 1,614 participants who completed both enrollment visits between March 2013 to August 2018, a total of 1,342 individuals underwent HIV testing and completed questions on oral sex practices and other significant covariates. One hundred sixty-two (162, 10%) individuals were excluded because of missing HIV status, 22 (1%) were missing oral sex practices, and 88 (5%) were missing covariate data necessary in the final model. The prevalence of oral sex was 69% (95% CI: 66–71) and the prevalence of HIV infection was 58% (95% CI: 56–60). Seventy-eight percent of individuals living with HIV identified as men, and 85% ever tested for HIV during their lifetime. For those living with HIV, 87% engaged in receptive anal sex and 84% engaged in insertive anal sex. HIV prevalence increased as number of male sexual partners increased, however HIV prevalence decreased with two or more female partners (Table 1).

**Table 1:** Demographic and behavioral characteristics of Nigerian MSM and TGW stratified by HIV status.

Factors	Living with HIV (n, col %) n = 776	At Risk of HIV (n, col %) n = 566	P-value^†^
Oral Sex*			**<0.01**
No	192 (25)	230 (41)	
Yes	584 (75)	336 (59)	
Gender Identity			**<0.01**
Man	605 (78)	486 (86)	
Woman	106 (14)	43 (8)	
Non-binary	65 (9)	37 (6)	
Mobile phone owned			**<0.01**
No	19 (2)	41 (7)	
Yes	757 (98)	525 (93)	
Alcohol use**			0.77
No	384 (50)	281 (51)	
Yes	380 (50)	269 (49)	
Condomless anal sex***			0.07
No	254 (33)	212 (38)	
Yes	520 (67)	351 (62)	
Prior HIV test			**<0.01**
No	117 (15)	149 (26)	
Yes	659 (85)	417 (74)	
Receptive anal sex*			**<0.01**
No	104 (13)	226 (40)	
Yes	672 (87)	340 (60)	
Insertive anal sex*			**<0.01**
No	200 (26)	93 (16)	
Yes	574 (74)	471 (84)	
Male anal partners*			**<0.01**
0–1	58 (8)	77 (13)	
2–4	274 (35)	225 (40)	
5+	444 (57)	264 (47)	
Female vaginal sexual partners*			**<0.01**
0–1	598 (77)	315 (56)	
≥2	178 (23)	251 (44)	
Transactional sex*			**0.01**
No	434 (56)	276 (49)	
Yes	342 (44)	290 (51)	

Abbreviations: OR, odds ratio; CI, confidence interval; MSM, men who have sex with men; TGW, transgender women.

^†^Pearson’s chi-square test. **Bolded** indicates p<0.05.

*Sexual activity and partner variables assessed in the past 12 months.

**Alcohol use assessed in the past 30 days.

***Condom use reported for most recent anal sex act with a male partner.

Seventy-five percent (95% CI: 73–78) of those living with HIV and 59% (95% CI: 56–63) of those at risk of HIV reported having oral sex (p<0.01). A larger proportion of those living with HIV reported more oral sexual partners in the last 12 months [0 to 1 males (36%), 2 to 4 males (31%), 5+ males (33%)] as compared to those who were not [0 to 1 males (53%), 2 to 4 males (27%), 5+ males (21%)] (p<0.01). Among men living with HIV, 96% reported any condomless oral sex in the last 12 months, while 4% reported always using a condom during oral sex. Similarly, 93% of those not living with HIV reported any condomless oral sex in the last 12 months and only 7% reported always using a condom. Additionally, those who self-identified as women, owned a mobile phone, had a prior HIV test, and engaged in transactional sex were more likely to report oral sex practices (Table 2). Seventy-four percent of those who had receptive anal sex also reported oral sex and 68% of those who had reported insertive anal sex also reported oral sex. As number of male partners increased the proportion of those who had oral sex also increased [0 to 1 males (50%), 2 to 4 males (62%), 5+ males (77%)]. As female partner number increased, the proportion of those who had oral sex decreased [0 to 1 females (72%), ≥2 females (61%)].

**Table 2:** Demographic and behavioral characteristics associated with oral sex among MSM and TGW in Nigeria.

Factors	Oral sex (n, row %) n = 920	No oral sex (n, row %) n = 422	OR (95% CI)	aOR^†^ (95% CI)
HIV status				
At risk	336 (59)	230 (41)	Ref.	Ref.
Living with	584 (75)	192 (25)	**2.1 (1.6–2.6)**	**1.4 (1.1–1.8)**
Gender Identity				
Man	722 (66)	369 (34)	Ref.	Ref.
Woman	122 (82)	27 (18)	**2.1 (1.4–3.3)**	**1.8 (1.1–2.8)**
Non-binary	76 (75)	26 (25)	1.4 (0.9–2.3)	1.2 (0.7–2.0)
Mobile phone owned				
No	29 (48)	31 (52)	Ref.	Ref.
Yes	891 (69)	391 (31)	**2.4 (1.5–4.1)**	**2.3 (1.3–3.9)**
Alcohol use**				N/A
No	427 (64)	238 (36)	Ref.	
Yes	481 (74)	168 (26)	**1.6 (1.3–2.0)**	
Condomless anal sex***				
No	338 (73)	128 (27)	Ref.	N/A
Yes	578 (66)	293 (34)	**0.8 (0.6–1.0)**	
Prior HIV test				
No	159 (60)	107 (40)	Ref.	Ref. **1.6 (1.2–2.2)**
Yes	761 (71)	315 (29)	**1.6 (1.2–2.1)**	
Receptive anal sex*				
No	176 (53)	154 (47)	Ref.	Ref.
Yes	744 (74)	268 (26)	**2.4 (1.9–3.1)**	**1.7 (1.3–2.3)**
Insertive anal sex*				N/A
No	207 (71)	86 (29)	Ref.	
Yes	711 (68)	334 (32)	0.9 (0.7–1.2)	
Male anal sexual partners*				
0–1	68 (50)	67 (50)	Ref.	Ref.
2–4	307 (62)	192 (38)	**1.6 (1.1–2.3)**	**1.5 (1.0–2.2)**
5+	545 (77)	163 (23)	**3.3 (2.3–4.8)**	**2.9 (1.9–4.3)**
Female vaginal sexual partners*				
0–1	658 (72)	255 (28)	Ref.	Ref.
≥2	262 (61)	167 (39)	**0.6 (0.5–0.8)**	**0.7 (0.5–0.9)**
Transaction*				
No	466 (66)	244 (34)	Ref.	Ref.
Yes	454 (72)	178 (28)	**1.3 (1.1–1.7)**	1.2 (0.9–1.5)

Abbreviations: OR, odds ratio; CI, confidence interval; MSM, men who have sex with men; TGW, transgender women.

^†^Multivariable logistic regression model was adjusted for gender identity, mobile phone, prior HIV test, receptive anal sex male partners, and transaction. Alcohol, condomless anal sex, insertive anal sex were not determined to be significant confounders and were excluded from the model. **Bolded** indicates p<0.05.

*Sexual activity and partner variables assessed in the past 12 months.

**Alcohol use assessed in the past 30 days.

***Condom use reported for most recent anal sex act with a male partner.

### Evaluation of effect modification

After stratification by female partners, the association between HIV and oral sex practices did not significantly differ (0–1 partners, OR: 1.7, 95% CI 1.3–2.3; ≥2 female partners OR: 2.4, 95% CI: 1.6–3.7) and the stratified ORs were similar to the crude OR (OR: 2.1, 95% CI: 1.6–2.6) suggesting no multiplicative interaction (p = 0.2). No other variables were assessed for effect modification.

### Multivariable analysis

After adjusting for gender, mobile phone, prior HIV test, receptive anal sex, male partner number, female partner number, and transactional sex, reporting oral sex practices was 40% more likely among those living with HIV as compared to those who were not (Table 2). Women identifying participants had higher odds of reporting oral sex as compared to participants who identified as men. Other independent predictors included owning a mobile phone, prior HIV testing, and engaging in receptive anal sex. Those with more than 5 male partners reported higher odds of oral sex as compared to 4 or fewer. Those with more female partners reported a lower likelihood of oral sex with male partners. Alcohol use and condomless anal sex did not confound our main association and were not included in the final model.

### Oropharyngeal STI prevalence

Of 752 participants who were tested for oropharyngeal STIs, the overall prevalence of NG or CT was 6.9% (95% CI: 5.1–8.7). The prevalence of NG was 5.2% (95% CI: 3.6–6.8) and CT was 2.3% (95% CI: 1.2–3.3). Of those who reported oral sex partners or condom practices with oral sex, 8.6% (95% CI: 6.1–11.1) tested positive for an oropharyngeal STI as compared to 3.6% (95% CI: 1.3–5.9) of those who did not self-report oral sex practices. Oropharyngeal bacterial STIs were nearly 3-fold higher for those who reported oral sex practices as compared to those who did not (unadjusted OR = 2.5, 95% CI: 1.2–5.3) (Table 3).

**Table 3:** Association of oral sex and bacterial oropharyngeal STIs among MSM and TGW in Nigeria.

STIs	Any Oral Sex*	No Oral Sex	Crude OR**
	n (col%)	n (col%)	(95% CI)
	n = 500	n = 252	
Any Oropharyngeal STI	43 (8.6)	9 (3.6)	**2.5 (1.2, 5.3)**
Oropharyngeal Gonorrhea	31 (6.2)	8 (3.2)	2.0 (0.9, 4.4)
Oropharyngeal Chlamydia	15 (3.0)	2 (0.8)	3.9 (0.8, 1.1)

Abbreviations: STI, sexually transmitted infection; col, column; OR, odds ratio; CI, confidence interval; MSM, men who have sex with men; TGW, transgender women.

*Oropharyngeal STIs categorized as any oral *Neisseria gonorrhoeae* [NG] and/or *Chlamydia trachomatis* [CT].

**Unadjusted odds ratios calculated using logistic regression model. **Bolded** indicates p<0.05.

## Discussion

With nearly 70% of participants reporting oral sex practices with male partners, our estimate was within the range of previously reported estimates among reproductive-aged men in sub-Saharan Africa (8–84%) [4,16–18] and more similar to MSM in the United States (76–97%) [8, 19, 20]. MSM aged 14–39 years in Bangui, Central African Republic reported a higher estimate (100%) of receptive oral sex for those who engaged in sex with men-only and 86% for those living with HIV, although the limited sample size may have resulted in an overestimate in behavior [4]. For adolescent MSM app-users in the United States aged 14–17 years, the prevalence of oral sex was similar to our findings at 76% [19] and slightly lower than the estimates (86–97%) from two other studies in the US for MSM in their mid-twenties and thirties [8, 20]. Our data suggest Nigerian MSM compared to US MSM frequently reported 5 or more multiple oral sexual partners (28% vs. 17%, respectively) [20]. Overall, oral sex practices among MSM and TGW in Nigeria fall within the range of prior estimates but evaluating consistency is somewhat limited by the number of studies, differences in the type of data captured, and mode of data collection (in-person interviews versus apps and internet surveys) [31, 32].

Multiple sexual partners were independently associated with a 2-fold higher odds of oral sex, as has been previously described in the United States and China [6, 33, 34]. Our data expands on prior work, as it demonstrates an increased odds of oral sex with increasing number of male anal sexual partners, and decreased odds of oral sex with two or more female sexual partners independent of HIV status. Oral sex has been reported to be less common among MSMW as compared to MSM in several other settings [8,35–37]. In a study in the Southeastern United States, MSMW were 80% less likely to have 2 or more male oral sexual partners as compared to MSM-only [37]. At an STI clinic in the US, oral sex practices were 8-fold lower for MSMW as compared to MSM-only (10% vs. 87%, respectively) [8]. Similarly, self-identifying bisexual MSM reported an 8-fold lower estimate of oral sex at their last sexual encounter as compared to gay-identifying MSM (13% vs. 87%, respectively) [36]. Many of these prior studies did not assess oral sex practices independent of HIV infection, which may have confounded the association between oral sex and partner type. Although, our data suggest gender of sexual partners may be related to the frequency of behavior.

Prior studies found HIV testing behavior was significantly associated with sexual practices, but anal sexual practices were pooled with oral under an all-inclusive category [38–40] or the studies only evaluated anal sex [41, 42]. In Cameroon, prior HIV testing was associated with having 4 or more oral or anal sexual partners in the past year as compared to 1 to 3 sexual partners, but this association was not stratified by sexual practice [40]. In another study, individuals who knew their HIV status and received counseling were more likely to adopt less risky sexual practices, but the authors did not define what sexual practices were less risky [42]. In an analysis evaluating the impact of testing on behavior, self-reported oral sex practices increased from 57% to 79% before and after HIV testing among Chinese MSM not living with HIV [43]. Longitudinal studies including TRUST/RV368 could evaluate whether HIV testing leads to an uptake of oral sex practices.

Our findings suggest other independent correlates of oral sex include gender identity and owning a mobile phone. Women-identifying participants also reported a higher odds of oral sex, consistent with a quantitative study in Thailand that suggested TGW were more likely than MSM to prefer receptive anal sex and engage in only oral sex [44]. Moreover, our study identified an association between mobile phone ownership and oral sex, a sexual practice that has not been differentiated from anal sexual practices in prior studies [45–47].

Many oropharyngeal bacterial STIs such as NG and CT are often asymptomatic [48–51] and estimates of their prevalence is limited by insufficient testing [52–54]. Our overall prevalence of oropharyngeal STIs was 7%, 5% for NG and 2% for CT which is consistent with estimates from higher-income countries. In Australia, oral NG was 3% for teenage MSM self-reporting both insertive and receptive oral sex with men [55]. In the United States, oral NG ranged from 1 to 17% and CT ranged from 0 to 4% as described in a systematic review of extragenital infections in MSM [48, 49]. In a London HIV care center serving MSM, the prevalence of oral NG and CT was 4% and 2%, respectively [56]. Our findings from Nigeria are comparable with studies from higher-income countries and suggest oral sex behaviors are associated with a 3-fold higher odds of oropharyngeal STIs. Although, oropharyngeal STIs are relatively uncommon as compared to STIs at urogenital and anorectal sites [23, 57], it is important to increase STI screening and condom use at all sites to reduce the overall population burden.

This study has some limitations. The questionnaires surveyed participants about sexual behaviors in the past 12 months, making reporting susceptible to recall bias. Although, asking specifically about the number of oral sex partners and condom use with oral sex gave structure to the participants in order to minimize reporting bias on oral sex practices. Moreover, testing for oral bacterial STIs was initiated later in the study, limiting our estimates for the entire cohort. However, in comparison to most studies our sample size for oropharyngeal STI testing is still considerably large with over 700 participants with laboratory diagnoses rather than self-report. Additionally, we were limited in temporality because of the cross-sectional design, but utilized behavioral questions with time components to attempt to capture sexual behavior over a year-long period. This cohort draws from two large cities in Nigeria and therefore may not be generalizable to other areas or on a national scale. Lastly, we were unable to differentiate insertive and receptive oral sex. However, our findings established the frequency of this behavior that will support future work investigating specific oral sex positions.

## Conclusions

Oral sex practices were high among Nigerian MSM and TGW and associated with an increased odds of oropharyngeal STIs. Without clear guidelines on screening and treatment of oropharyngeal STIs, our data reinforce the importance of condom messaging for oral sex together with other sexual practices. This is particularly important for bacterial STIs that are clinically asymptomatic and continue to impact the health of Nigerian MSM and TGW. Future work could evaluate biomedical pre-exposure prophylaxis for bacterial STIs to complement current prevention strategies.

## Supporting information

S1 Questionnaire (PDF)

S2 Questionnaire (PDF)
